# Fit to Perform: An Investigation of Higher Education Music Students’ Perceptions, Attitudes, and Behaviors toward Health

**DOI:** 10.3389/fpsyg.2017.01558

**Published:** 2017-10-10

**Authors:** Liliana S. Araújo, David Wasley, Rosie Perkins, Louise Atkins, Emma Redding, Jane Ginsborg, Aaron Williamon

**Affiliations:** ^1^Centre for Performance Science, Royal College of Music, London, United Kingdom; ^2^Faculty of Medicine, Imperial College London, London, United Kingdom; ^3^Cardiff School of Sport, Cardiff Metropolitan University, Cardiff, United Kingdom; ^4^Trinity Laban Conservatoire of Music and Dance, London, United Kingdom; ^5^Royal Northern College of Music, Manchester, United Kingdom

**Keywords:** coping, fatigue, health promotion, music, perfectionism, performance, sleep, wellbeing

## Abstract

Making music at the highest international standards can be rewarding, but it is also challenging, with research highlighting pernicious ways in which practicing and performing can affect performers’ health and wellbeing. Several studies indicate that music students’ perceptions, attitudes, and behaviors toward health and healthy living are less than optimal, especially considering the multiple physical and psychological demands of their day-to-day work. This article presents the results of a comprehensive screening protocol that investigated lifestyle and health-related attitudes and behaviors among 483 undergraduate and postgraduate students (mean age = 21.29 years ± 3.64; 59% women) from ten conservatoires. The protocol included questionnaires measuring wellbeing, general health, health-promoting behaviors, perfectionism, coping, sleep quality, and fatigue. On each measure, the data were compared with existing published data from similar age groups. The results indicate that music students have higher levels of wellbeing and lower fatigue than comparable samples outside of music. However, they also reveal potentially harmful perceptions, attitudes, and behaviors toward health. Specifically, engagement in health responsibility and stress management was low, which along with high perfectionistic strivings, limited use of coping strategies, poor sleep quality, and low self-rated health, paints a troubling picture both for the music students and for those who support their training. The findings point to the need for more (and more effective) health education and promotion initiatives within music education; in particular, musicians should be better equipped with mental skills to cope with constant pressure to excel and high stress levels. In part, this calls for musicians themselves to engage in healthier lifestyles, take greater responsibility for their own health, and be aware of and act upon health information in order to achieve and sustain successful practice and performance. For that to happen, however, music educators, administrators, and policy makers must play an active role in providing supportive environments where health and wellbeing is considered integral to expert music training.

## Introduction

Pain, musculoskeletal problems, and performance anxiety are prevalent among musicians, and these manifestations of ill-health impact considerably on musicians’ performance, as well as on their career progression and general wellbeing (Caldron et al., 1986; Fishbein et al., 1988; Zaza, 1998; Bragge et al., 2006; Kenny and Ackermann, 2015). The research into musicians’ health undertaken over the past three decades has employed mainly clinical and diagnostic approaches to identifying and understanding the multitude of problems that arise from music practice and performance, in particular in the Western classical tradition. This research has made great strides toward legitimizing the health challenges that classical musicians face and toward enabling discussion of health issues both among musicians and more widely within educational and professional contexts (Brandfonbrener, 1986; Jabusch et al., 2004; Altenmüller and Jabusch, 2009; Ackermann et al., 2012, 2014). Indeed, health education and provision has increased considerably within music in the intervening years (for reviews, see Chesky et al., 2006; Williamon and Thompson, 2006), although not nearly enough to match the size and scope of problems reported in the literature and driven by an agenda that is predominantly reactive rather than preventative and proactive.

By contrast, existing models of health promotion advocate that an individual’s engagement in actions that prevent ill-health and improve wellbeing results from a reciprocal relationship between his or her characteristics, previous experiences, and environmental influences (Nutbeam, 2000; Pender, 2011). Engaging in a healthy lifestyle is thus crucial for ensuring that performers are in top physical and mental condition to meet the demands of making music, preventing ill health and optimizing their performance. In studies of musicians’ health, professional classical musicians often report a lack of preparation in their early years of training for anticipating and dealing effectively with the challenges and strains of the profession (Wynn Parry, 2004). At present, it is still unclear whether music students are developing the skills and strategies during their music training to sustain good health and to cope effectively with the demands of the profession. In our recent qualitative study on the barriers and enablers to optimal health (Perkins et al., 2017), music students commented on the daily challenges that they faced in sustaining a healthy lifestyle, along with the demands arising from practice and performance. While the students recognized the value of health and believed in their ability to influence positively their wellbeing, research points to a gap between students’ perceptions and beliefs versus their actions (Spahn et al., 2004).

It is essential, therefore, to understand the health profiles of music students at early stages in their careers to understand better their specific health needs and identify areas for action to prevent physical and psychological problems. Moreover, while acknowledging that music students in specialist higher education face specific challenges (e.g., constant scrutiny, pressure to excel, and intense competition), they also share many of the same difficulties experienced by peers studying in other subjects. These include adjusting to new environments and to levels of study that demand advanced learning skills and specialist knowledge, while also facing new personal, social, and financial constraints that may impact on their health, wellbeing, and performance (Vaez and Laflamme, 2003; Spahn et al., 2004; Brown and Schutte, 2006; Mikolajczyk et al., 2008; Davoren et al., 2013). Healthy lifestyles and behaviors have thus been a major concern within higher education institutions as key determinants of quality of life, wellbeing, and health status.

Existing research suggests that music students engage very poorly in health-promoting behaviors (Spahn et al., 2002, 2004; Kreutz et al., 2008, 2009; Ginsborg et al., 2009; Panebianco-Warrens et al., 2015; Rickert et al., 2015), but further comprehensive study of musicians’ health perceptions, attitudes, and behaviors is needed. As a result, Conservatoires UK launched *Musical Impact* in 2014, a 4-year research project aimed at investigating the health and wellbeing of musicians working and studying in the United Kingdom. The project has three core strands: (1) Fit to Perform explores the attitudes, perceptions, and behaviors of musicians toward health and wellbeing, as well as their experience of chronic and acute health problems and their general fitness for performance; (2) Making Music investigates the physical and mental demands faced by musicians as they practice and perform; and (3) Better Practice examines strategies for promoting health effectively in music educational and professional contexts. This article arises from Fit to Perform and addresses the wellbeing and health-related perceptions, attitudes, and behaviors of higher education music students. Specifically, we report on wellbeing, self-rated health, health-promoting lifestyles, coping, perfectionism, sleep quality, and fatigue, given the relevance of these constructs to health and wellbeing among university students more generally.

Wellbeing has been extensively researched as an indicator of quality of and satisfaction with life and optimal functioning, and it has been associated with both physical and mental health (Ryan and Deci, 2001; Chanfreau et al., 2008; Davoren et al., 2013; Prendergast et al., 2016). In the general population (Booker and Sacker, 2011; Davoren et al., 2013; Office for National Statistics, 2016), studies have shown that women tend to score lower on wellbeing than men, a pattern that has not yet been fully explained. One possible explanation is that women and men attribute different descriptors to wellbeing and, therefore, develop different perceptions of it (Singletary et al., 2014). Recent studies in music have shown that, despite the highly demanding contexts that musicians face in educational and professional realms, they find high levels of meaning in their lives and experience feelings of accomplishment (Kivimäki and Jokinen, 1994; Ascenso et al., 2017). Research also shows that musicians’ satisfaction with life, as well their levels of positive and negative affect, are associated with setting clear goals, autotelic experiences, and an optimal balance between challenge and skill (Fritz and Avsec, 2007; Bonneville-Roussy et al., 2011), comparable to that found in university students in other subjects (Kiaei and Reio, 2014).

Outside of music, the association between wellbeing, health perceptions and behaviors, and academic performance has been widely investigated in higher education settings, with associations identified with sleep, fatigue, stress, and physical and emotional health (Stewart et al., 1992; Stewart-Brown et al., 2000; Steptoe et al., 2006; Donders et al., 2007; Maghout-Juratli et al., 2010). Findings indicate that the self-rated health of university level students is generally poor and strongly associated with emotional distress and psychosomatic complaints, as well as financial concerns, which may impact their general wellbeing (Roberts et al., 2000; Stewart-Brown et al., 2000; Shields and Shooshtari, 2001; Steptoe et al., 2006; Mikolajczyk et al., 2008). In addition, research suggests that university students show poor to moderate engagement in healthy behaviors, which has led to health promotion initiatives worldwide such as Healthy Campus 2020 in the United States (American College Health Association, 2012) and Healthy Universities in the United Kingdom^1^. Similar findings have been observed in musicians, who seem to display poor health awareness, unrealistic expectations of finding external answers to their health problems, and low engagement in health responsibility and stress management (Kreutz et al., 2009; Panebianco-Warrens et al., 2015; Rickert et al., 2015).

Considering that stress is highly prevalent among others in higher education (Robotham and Julian, 2006), the way individuals deal with taxing events and use their coping resources is crucial and may impact on their health and wellbeing (Lazarus, 1993; Taylor and Stanton, 2007), as well as on academic performance (Robotham and Julian, 2006). Despite the wealth of research into coping, aspects related to age and sex differences in coping in adulthood, and in particular in young adults, remain unclear. Coping changes with age, with increased ability observed in older adults, but how it develops and changes depend on factors such as vulnerability, exposure, life events, and life roles (Aldwin, 2011). There may also be an effect of sex in coping that is also mediated by multiple variables, such as perceptions of health, stress reactivity, and gender roles (Ptacek et al., 1994; Matud, 2004; Helgeson, 2011). As to research on musicians’ coping strategies, a limited number of studies were published in the late 1980s reflecting a growing interest in musicians’ health (Steptoe and Fidler, 1987; Dews and Williams, 1989; Steptoe, 1989). Subsequent research has begun to examine links between coping and aspects of health and wellbeing (Kobori et al., 2011; Biasutti and Concina, 2014; Braden et al., 2015); the findings suggest that breathing, relaxation techniques, positive reframing, and task-oriented coping, as well as medication, are among the most used coping strategies by musicians. Dysfunctional coping using social support and avoidance strategies appear to be related to music performance anxiety (MPA), which can be explained by the social judgment dimension of MPA.

Often linked to increased pressure and stress is perfectionism. Perfectionistic strivings are associated with positive characteristics, behaviors, and outcomes, while perfectionistic concerns are associated with negative characteristics, behaviors, and outcomes (Frost et al., 1993; Stoeber and Otto, 2006; Stoeber and Eismann, 2007; Stoeber et al., 2007, 2009; Stoeber, 2012; Harrison and Craddock, 2016). Perfectionism has been reported as common among musicians, but systematic research on perfectionism in music is limited and the evidence is mixed (Sinden, 1999; Stoeber and Eismann, 2007). A study by Kenny et al. (2004) revealed associations between perfectionism and general anxiety, MPA, and coping resources, although without predictive value. However, Kenny’s study did not clarify the specific interaction between different facets of perfectionism. Nevertheless, the limited number of studies on perfectionism in musicians indicate that perfectionistic concerns are associated with MPA, external motivation, and other forms of distress, while perfectionistic strivings are associated with successful achievement and positive characteristics (Sinden, 1999; Stoeber and Eismann, 2007).

Stress also impacts on lifestyle behaviors. For instance, the main complaints of poor sleep among university students are associated with emotional and academic stress more than sleep practices, and these impact on their psychological health (Brown et al., 2002; Carney et al., 2006; Lund et al., 2010; Orzech et al., 2011). Sleep quality is important not only for body homeostasis and consequent health and wellbeing but also for learning and memory consolidation (Harrison, 2011; Wheaton et al., 2016). Evidence shows that sleep quality improves motor skill learning and memory in simple tasks, and the gains of sleep are the highest as memory load and motor complexity increase (Walker et al., 2002; Kuriyama et al., 2004; Appleman et al., 2016). Several studies have shown that poor sleep quality and risk of sleep disorders are common in early adulthood, especially among university students (Zeitlhofer et al., 2000; Brown et al., 2002; Lund et al., 2010; Orzech et al., 2011; Wolfson and O’Malley, 2012; Chang et al., 2016; Wheaton et al., 2016). As to risk factors associated with poor sleep quality and poor sleep hygiene (i.e., sleep habits and practices that affect sleep quality), research points to high alcohol and caffeine intake, erratic schedules, environmental noise (especially for those sleeping in university residences), stress and worrying (Brown et al., 2002, 2009; Carney et al., 2006; Lund et al., 2010; Wheaton et al., 2016). The literature also suggests that knowledge about sleep hygiene does not necessarily have a direct impact on sleep quality, but it can lead to change in sleep practices and behaviors, which in turn will improve sleep quality (Brown et al., 2002). Research on sleep and its implications for music students is scarce. Vaag et al. (2015) investigated sleep patterns of the Norwegian workforce and concluded that professional musicians had a higher prevalence of insomnia symptoms than the general workforce due to non-restorative sleep and dissatisfaction with sleep.

Poor sleep, drinking behaviors, and stress have also been investigated in association with chronic fatigue syndrome but only few studies of non-clinical samples of university students have been undertaken (Pilcher et al., 1997; Alapin et al., 2000; Brown and Schutte, 2006; Tanaka et al., 2009). Fatigue can be defined in physiological terms, as muscle exhaustion, or in behavioral terms, as a decrement in performance and subjective feelings of tiredness and weakness (Chalder et al., 1993). Debilitating levels of fatigue can affect individuals’ performance in their daily lives, and when fatigue occurs alongside sleep disorders, pain, and cognitive impairment, it can develop into a chronic condition (Jackson and MacLeod, 2016).

Altogether, the existing research points to a multitude of factors that may impact students’ general health and wellbeing. Research has consistently shown that music students have overall poor engagement in healthy lifestyles, in particular health responsibility and stress management. However, only a limited number of studies have addressed the coping strategies of music students, their perfectionism levels, or their self-rated health. Moreover, health-related topics such as sleep and fatigue remain under investigated among musicians: to our knowledge, only one study has addressed sleep quality of professional musicians (Vaag et al., 2015), and most studies have focused on muscular fatigue (Chan et al., 2000; Drinkwater and Klopper, 2010; Hildebrandt et al., 2012), with little corresponding work on fatigue defined behaviorally.

This article presents new findings of a comprehensive investigation into lifestyle and health-related attitudes and behaviors of higher education music students, mainly studying in the Western classical tradition. Constructs such as self-rated health, lifestyle behaviors, coping, perfectionism, fatigue, and sleep have been widely associated with health and wellbeing of higher education students and have been shown to be critical in understanding the health attitudes, perceptions, and behaviors of this specific age group. Music students’ health and wellbeing are often investigated in relation to the specific challenges of being a musician, but to date, no other study has explored these key health-related constructs in a comparative and comprehensive way. This article aims to provide a health profile of music students in relation to their peers in higher education, where possible, and/or to normative data. This study takes an important step in generating an evidence base for the development of health education and health promotion initiatives, with the aim of describing, understanding, and enhancing the health and wellbeing of musicians from early stages of their careers. By doing so, we intend to position health and wellbeing as a driver, rather than the consequence of, music making and performance enhancement.

## Materials and Methods

### Participants

483 musicians (286 women, 197 men) studying in higher education were recruited in person and by email from ten conservatoires, nine in the United Kingdom and one in southern Switzerland, over a period of 9 years (2006–15). 42% of participants (*n* = 204) reported their nationality, of whom 42% were British (*n* = 86), 21% Italian (*n* = 42), and the remaining 37% from 30 other countries. The mean age of the sample was 21.3 years (SD ± 3.64), 21.44 years for women (±3.74, range 17–51) and 21.06 years for men (±3.48, range 17–41). Sample characteristics including instrumental group, primary performance genre, and year and institution of study are provided in **Table 1**. At the time of participation, 322 were undergraduate students, and 161 were postgraduate students. Most participants (95%) identified themselves as classical musicians, with the remaining 5% identifying mainly with pop, jazz, or folk genres.

**Table 1:** Number of women and men according to instrument group, primary performance genre, and year and institution of study.

	Women *n* = 286 (59%)	Men *n* = 197 (41%)	Totals *N* = 483	%
**Instrument group**				
Strings	110	64	174	36%
Keyboard	51	45	96	20%
Woodwind	66	27	93	19%
Brass	12	28	40	8%
Voice	38	11	49	10%
Percussion	6	8	14	3%
Other	3	14	17	4%
				100%
**Performance genre**				
Classical	267	190	457	95%
Non-classical (pop, jazz, folk)	19	7	26	5%
				100%
**Year of study**				
Undergraduate (UG) year 1	131	102	233	48%
UG year 2	14	19	33	7%
UG year 3	15	16	31	6%
UG year 4	15	10	25	5%
Postgraduate (PG) year 1	77	33	110	23%
PG year 2	26	13	39	8%
PG other	8	4	12	3%
				100%
**Institution of study**				
Birmingham Conservatoire (United Kingdom)	10	4	14	3.0%
Conservatorio della Svizzera Italiana (Switzerland)	35	31	66	13.7%
Guildhall School of Music and Drama (United Kingdom)	4	0	4	0.8%
Leeds College of Music (United Kingdom)	2	3	5	1.0%
Royal Central School of Speech and Drama (United Kingdom)	17	2	19	3.9%
Royal College of Music (United Kingdom)	149	114	263	54.5%
Royal Conservatoire of Scotland (United Kingdom)	10	6	16	2.9%
Royal Northern College of Music (United Kingdom)	49	31	80	16.6%
Royal Welsh College of Music and Drama (United Kingdom)	6	4	10	2.1%
Trinity Laban Conservatoire of Music and Dance (United Kingdom)	4	2	6	1.2%
				100%


**Strings:* violin, viola, viola de Gamba, cello, double bass, guitar (classical and electric), and harp; *Keyboard:* accordion, piano, organ, harpsichord, and historical keyboards; *Woodwind:* flute, recorder, clarinet, oboe, bassoon, and saxophone; *Brass:* cornet, euphonium, horn, trombone, trumpet, and tuba; *Other:* composition and conducting.*

Existing published data (mean values) were used for comparisons with the broader higher education student population. When unavailable, comparisons were made using published data for the general population (mainly United Kingdom, where the majority of the sample was based) using data from the same age range. Clarifications of specific comparisons are provided below on a variable-by-variable basis.

### Procedure

**FIGURE 1:**
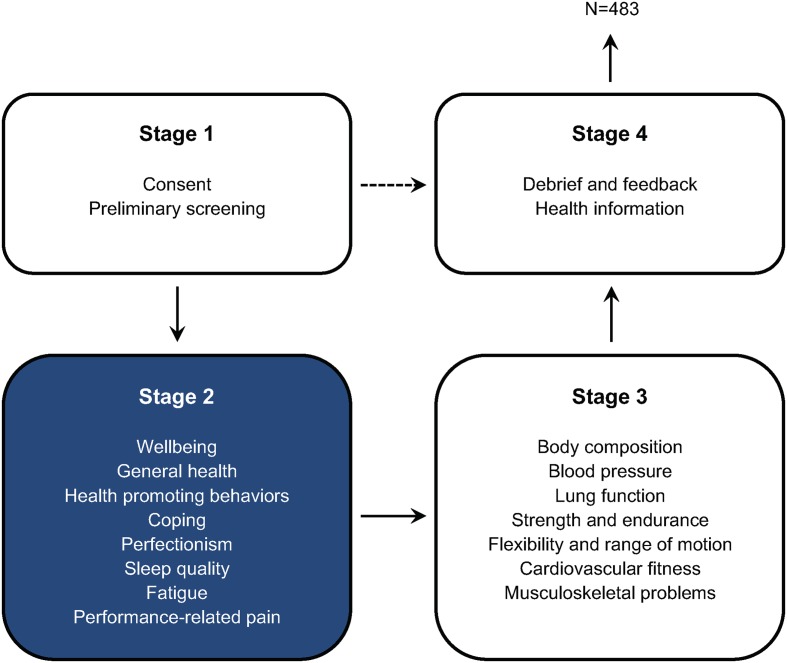
Flow of participants involved in the Fit to Perform screening protocol. This article focuses on a selection of measures from Stage 2 (*N* = 483), a survey of the perceptions, attitudes, and behaviors of music students toward health and wellbeing. 32 of 515 prospective participants were excluded from analyses. For a complete list of measures used in the Fit to Perform protocol, see Supplementary Table 1.

The Fit to Perform screening protocol was developed as a physical and mental health assessment package for musicians, first compiled in 2006 and then expanded and refined in 2013. Component measures were drawn from those employed in previous studies shown to be pertinent for musicians’ health (Tsigilis et al., 2002; Vanhees et al., 2005; Ackermann and Driscoll, 2010), as well as other standardized measures deemed relevant for addressing the project’s research questions. At each stage of development, the protocol was piloted among members of the research team and with a small number of music students to check timings and to elicit feedback on the suitability of measures. Prior to participation, each musician was sent an information sheet that included instructions on alcohol, caffeine, and food intake prior to the assessment (Hoffman, 2006). Assessments were conducted with individual musicians and consisted of four stages (see **Figure 1** and Supplementary Table 1):

1. *Stage 1* (5 min): introductory briefing for the participant, collecting signed informed consent from the participant, and a preliminary screening using the Physical Activity Readiness-Questionnaire (PAR-Q) to assess participants’ suitability for taking part in a sub-maximal cardiovascular fitness test (ACSM, 2014).
2. *Stage 2* (40 min): survey, delivered online using Surveymonkey^®^, including self-report measures of health-promoting behaviors, perceptions, and attitudes to health and wellbeing based on existing questionnaires (see section ‘Stage 2 Measures’ below). Participants also completed a pain drawing on a digital interface (Apple iPad 2) using a stylus pen designed for tablets (CS100B, Wacom, Vancouver, WA, United States) and a commercially available sketching software (SketchBook Pro) (Barbero et al., 2015; Cruder et al., 2017).
3. *Stage 3* (35 min): assessment of body composition (i.e., height, weight), resting blood pressure, lung function, strength and endurance (i.e., hand grip, plank and press-up), flexibility and range of motion, and cardiovascular fitness. Participants completed a version of the Nordic Musculoskeletal Questionnaire–Extended (Dawson et al., 2009). Where pain in the arm, shoulder, and hand was reported, they also completed the Quick Dash (Germann et al., 1999; Beaton et al., 2005).
4. *Stage 4* (10 min): debriefing and summary of a selection of Stage 3 fitness results (i.e., blood pressure, cardiovascular fitness, grip strength, sit and reach, and press up) where data were immediately processed and could be compared against published norms, as well as providing relevant health-promotion leaflets and health education information.

Each assessment was allocated 90 min in total and was facilitated by at least three members of the research team trained to follow the detailed protocol consistently when administering the set measures. Assessments took place at each of the participating conservatoires at a pre-arranged date and time. Ethical approval for the research was granted by an independent sub-committee of the Conservatoires UK Research Ethics Committee.

### Stage 2 Measures

In the first part of the Stage 2 survey, information on musical experience and personal background was collected, including age, sex, year of study, and primary instrument. The subsequent sections of the survey consisted of standardized questionnaires on health, wellbeing, and psychological variables as detailed below.

#### Wellbeing

Wellbeing is a complex concept that refers to a sense of optimal equilibrium between personal challenges and resources, as well as the effective management of positive and negative affect, in order to achieve meaning in and satisfaction with life (Ryan and Deci, 2001; Dodge et al., 2012). To measure wellbeing, we used the Short Warwick-Edinburgh Mental Wellbeing Scale (SWEMWBS; Stewart-Brown et al., 2009), a 7-item questionnaire that assesses both hedonic (e.g., subjective experiences of happiness and life satisfaction) and eudaimonic (e.g., positive psychological functioning, good relationships, and self-realization) aspects of optimal psychological functioning (Tennant et al., 2007; Stewart-Brown et al., 2009).

Participants reported their feelings and thoughts over the preceding 2 weeks by responding to statements on 5-point scales from 1 (none of the time) to 5 (all of the time). The sum of the items yields a raw score between 7 and 35, with higher scores indicating greater mental wellbeing. The raw scores are then converted into an interval scale score for parametric analyses as recommended by Stewart-Brown et al. (2009). The SWEMWBS displays good psychometric properties (i.e., unidimensionality, freedom of item bias, and internal consistency) and is a suitable measure for use when time and participant fatigue is of concern. In our study, the internal reliability of the scale was acceptable (Cronbach α = 0.76).

#### General Health

Health perceptions refer to personal beliefs and evaluations of general health (Stewart et al., 1992). Self-rated health is a widely used indicator of quality of life, health-promoting behaviors, and individual use of health services in population studies (Hunt et al., 1985; Brazier et al., 1992; WHOQOL Group, 1995; McDowell, 2006; Jylhä, 2009; Pender, 2011). Research suggests that self-rated health varies according to age, sex, cultural background, comparisons with others, prior experiences, and life events (Wardle et al., 2004; McDowell, 2006; Jylhä, 2009).

Perceptions of general health are usually measured through a single item or a very small number of items answered in scales ranging from excellent to poor, as is the case of the general health scale of the RAND Short Form 36 Health Survey used here (SF-36; Ware and Sherbourne, 1992; McDowell, 2006). This scale consists of five items answered on a 5-point scale. One of the items is answered from 1 (excellent) to 5 (poor), and the remaining four are answered from 1 (definitely true) to 5 (definitely false). Answers are recoded to values of 100, 75, 50, 25, and 0. Scores represent a percentage of a total possible score achieved, and higher scores indicate a more favorable health state. This measure has shown good reliability coefficients in several studies (McDowell, 2006), with an acceptable internal reliability of α = 0.73 in the current study.

#### Health-Promoting Behaviors

Health-promoting behaviors refer to those actions that are part of one’s daily pattern of living, over which the individual has control, and that influence one’s health status and quality of life (Walker et al., 1987, 1995). The Health Promoting Lifestyle Profile II (HPLP II; Walker and Hill-Polerecky, unpublished) is a 52-item questionnaire that measures the extent to which individuals engage in six dimensions of a health-promoting lifestyle and has been used widely to investigate behaviors of tertiary level students across different fields of study. These dimensions are grounded in Pender’s health promotion model (Pender, 2011), a paradigm that advocates that individuals tend toward actions to decrease the probability of facing illness and to improve their wellbeing and self-actualization. This model also assumes that health-promoting behaviors result from a reciprocal relationship between the environment and individuals’ characteristics, experiences, and prior behavior.

The six dimensions are: health responsibility (HR, 9 items), physical activity (PA, 8 items), nutrition (NU, 9 items), spiritual growth (SG, 9 items), interpersonal relations (IR, 9 items), and stress management (SM, 8 items). Participants rated each item on a 4-point scale from 1 (never) to 4 (routinely). Total and subscale scores are obtained by calculating the mean of the individual’s responses to items, resulting in scores of between 1 and 4, with higher scores indicating higher levels of engagement on each of the six dimensions. Internal reliability of the scale in the original study (Walker and Hill-Polerecky, unpublished) was α = 0.94, with alpha coefficients for the subscales ranging from 0.79 to 0.87. In our study, HPLP II showed good internal reliability for the total scale (α = 0.90) and the following for the subscales: HR α = 0.78, PA α = 0.78, NU α = 0.72, SG α = 0.80, IR α = 0.77, and SM α = 0.62. The internal reliability of the SM subscale was low, similar to that observed in previous studies (Kreutz et al., 2009; Panebianco-Warrens et al., 2015). This subscale addresses both behavioral strategies (e.g., ‘practice relaxation or mediation’ for 15–20 min daily) and cognitive strategies (e.g., ‘concentrate on pleasant thoughts at bedtime’) to activate physical and psychological resources to control or reduce tension, and it may be the case that this is interfering with the dimensionality of the subscale. However, to allow comparisons with existing data, this subscale was used in our analysis.

#### Coping

Coping is an action-oriented effort to manage the demands of an event that is perceived as taxing in relation to one’s resources (Lazarus, 1993). Coping strategies have been categorized in many different ways, a major distinction being between emotion-focused coping (i.e., adjusting to the stressor) and problem-focused coping (i.e., changing the stressor) (Lazarus, 1993). While people can develop tendencies, or coping styles, in dealing with stressful events and use strategies consistently, they mainly adjust their coping strategies based on how the situation is appraised, the specific demands of the situation, and the personal resources available (Carver et al., 1989; Lazarus, 1993). To measure how music students cope with stressors, a situational version of selected scales of the COPE questionnaire (Carver et al., 1989) was used, where participants were asked to recall the strategies used in relation to the most recent stressful experiences. Participants indicated the degree to which they actually experienced each response during the last 7 days when facing a stressful experience. Each scale consists of 4 items rated on a 4-point scale from 1 (I didn’t do this at all) to 4 (I did this a lot). Scores are calculated by summing the value of each item, and the range is 4–16. Higher scores show higher use of coping strategies. The scales used (and the Cronbach alphas originally published for each) were: positive reinterpretation and growth (PRG, α = 0.68), focus on and venting of emotions (FVE, α = 0.77), active coping (AC, α = 0.62), planning (P, α = 0.80), suppression of competing activities (SCA, α = 0.68), use of instrumental social support (ISS, α = 0.75), and mental disengagement (MD, α = 0.45). The internal reliability of each scale in the current study was overall higher than in the original study with the exception of the MD subscale: PRG α = 0.65, FVE α = 0.83, AC α = 0.75, P α = 0.81, SCA α = 0.74, ISS α = 0.76, and MD α = 0.39. After removal of item ‘I turned to work or other substitute activities to take my mind off things’ in the MD scale, Cronbach alpha increased to 0.43, which was still very low. Therefore, the scale MD was not used in our analysis.

#### Perfectionism

Perfectionism refers to a personal trait characterized by setting exceedingly high standards (perfectionistic strivings) and tendencies for overcritical evaluations and negative reactions to mistakes (perfectionistic concerns) (Stoeber, 2012). These two facets of perfectionism (strivings and concerns) were measured using the Multidimensional Inventory of Perfectionism in Sports (MIPS; Stoeber and Eismann, 2007; Stoeber et al., 2007), comprised of two subscales: (1) striving for perfection (SP; 5 items) and (2) negative reactions to imperfection (NRI; 5 items). Participants indicated how they generally feel during performance (the statements remained unaltered as the concept of performance is also music-specific) on a 6-point scale from 1 (never) to 6 (always), and a mean for each subscale is calculated resulting in a score ranging from 1 to 6. Higher scores indicate higher perfectionistic strivings and more negative reactions to imperfection. The internal reliability of the scales in the current study was the same as that published by Stoeber and Eismann (2007): SP α = 0.92 and NRI α = 0.89. As per Stoeber’s (1998) recommendations, two subscales of the Frost Multidimensional Perfectionism Scale (‘concern over mistakes’ and ‘doubts about actions’) were also used to explore perfectionistic concerns further. The two subscales were merged into one subscale (CMD; Stoeber, 1998). CMD consists of 13 items where participants are asked how much they agree with the statements on a 5-point scale from 1 (strongly disagree) to 5 (strongly agree), resulting in score ranging from 1 to 5. Higher scores indicate higher perfectionistic concerns. The internal reliability of CMD in the current study was α = 0.92.

#### Sleep Quality

Sleep quality is a complex concept that includes quantitative aspects of sleep (e.g., duration, latency), as well as more subjective dimensions such as a feeling of restfulness or depth (Buysse et al., 1989). The Pittsburgh Sleep Quality Questionnaire (PSQI; Buysse et al., 1989) is a widely used measure of sleep quality assessed within a designated time frame (i.e., the last month). It consists of 19 self-rated questions that assess a variety of factors related to sleep quality grouped into seven component scores and equally weighted on a scale from 0 to 3: sleep quality, sleep latency, sleep duration, sleep efficiency, sleep disturbances, use of sleeping medication, and daytime dysfunction. These components are then summed resulting in a global score ranging from 0 to 21, where higher scores indicate poorer sleep quality. Buysse et al. (1989) suggest that a PSQI score greater than 5 is an indicator of sleep disturbance. The questionnaire has been shown to have good internal reliability (original α = 0.83) and good discriminant ability between ‘good’ and ‘bad’ sleepers (Buysse et al., 1989; Backhaus et al., 2002). In our study, the internal reliability was α = 0.62. Pearson correlations were performed to assess homogeneity of the scale (Carpenter and Andrykowski, 1998). Moderate correlations were significant between component scales and the total PSQI score at *p* ≤ 0.001. The lowest correlation with PSQI global score was with sleep medication (*r* = 0.39) and the highest was with sleep latency (*r* = 0.69). Poor correlations between sleep medication and the total score have been observed previously (Grandner et al., 2006) and may be related to the low use of medication in this sample (*M* = 0.12, *SD* = 0.43). As the total PSQI score requires all dimensions to be included, the sleep medication dimension was not removed, and the PSQI score was included in our analysis with cautious interpretations.

#### Fatigue

Fatigue is defined here using a behavioral approach (i.e., subjective feelings of weakness and tiredness; Chalder et al., 1993). It was measured using the Chalder Fatigue Questionnaire (CFQ; Chalder et al., 1993; Cella and Chalder, 2010), a short questionnaire consisting of 11 items (originally 14 items) answered on a 4-point scale from 0 (better than usual) to 3 (much worse than usual) to assess cognitive and physical symptoms of fatigue. A total score is calculated by adding the rating for each item. Total scores range from 0 to 33, with higher scores indicating higher levels of fatigue. This questionnaire has been widely used in community and clinical samples and has been shown to have good internal reliability (α = 0.89; Neuberger, 2003). In our study, the internal reliability was also good (α = 0.81).

### Data Treatment and Analyses

Data were analyzed using SPSS (v. 23). During data preparation, when less than 5% of individual answers per measure were missing, missing values were replaced with the individual mean value of the answers in each scale (or subscale) for each respondent. Outliers identified as having extreme *z*-scores of 3.25 or greater were removed from the dataset (Field, 2013). On the basis of the screening and after data preparation, 32 of 515 prospective participants were excluded from analyses, resulting in a final sample of 483 participants.

For comparisons with published normative or same-age group data, one-sample *t*-tests were calculated with mean values, and Cohen’s effect sizes were calculated. Hierarchical multiple linear regression procedures were used for each outcome variable (wellbeing, self-rated health, HPLP, coping, perfectionism, sleep, and fatigue) to investigate the effect of independent background variables (sex, level of study, instrument group). Linearity between variables was examined through scatterplots and reasonable linear relationships were observed with no extreme outliers. Confidence intervals of 95% were used in all analyses.

## Results and Discussion

Descriptive statistics for each measure are presented in **Table 2**. The results are reported on a variable-by-variable basis starting with comparisons with normative data (how music students compare with others), followed by within sample examinations (what may explain within sample results based on background variables such as sex, level of study and instrument group).

**Table 2:** Means, standard deviations, and t-statistics for all measures by sex.

Measure		*M*	*SD*	*t*	*p*	*d*
**Wellbeing (SWEMWBS)^1^**	W	22.43	2.95	-2.80	0.005	0.26
	M	23.21	3.04			
	Total	22.75	3.01			
**General health (GH)^2^**	W	60.98	18.57	-0.86	0.388	0.12
	M	63.20	16.63			
	Total	61.83	17.85			
**Health-promoting behaviors (HPLP II)^1^**						
Overall score	W	2.51	0.33	1.25	0.210	0.11
	M	2.47	0.35			
	Total	2.50	0.34			
Health responsibility (HR)	W	1.94	0.49	1.09	0.278	0.10
	M	1.89	0.51			
	Total	1.92	0.50			
Physical activity (PA)	W	2.22	0.55	-1.09	0.279	0.10
	M	2.28	0.63			
	Total	2.25	0.58			
Nutrition (NU)	W	2.69	0.51	3.62	<0.001	0.33
	M	2.52	0.52			
	Total	2.63	0.52			
Spiritual growth (SG)	W	2.87	0.49	-1.07	0.283	0.10
	M	2.91	0.49			
	Total	2.89	0.49			
Interpersonal relations (IR)	W	3.04	0.47	3.87	<0.001	0.35
	M	2.87	0.48			
	Total	2.97	0.48			
Stress management (SM)	W	2.26	0.40	-1.73	0.085	0.16
	M	2.33	0.43			
	Total	2.29	0.42			
**Coping (COPE)^2^**						
Positive reinterpretation and growth (PRG)	W	12.03	2.47	-0.51	0.069	0.07
	M	12.21	2.17			
	Total	12.10	2.35			
Planning (P)	W	11.58	2.92	1.18	0.241	0.16
	M	11.09	2.91			
	Total	11.40	2.92			
Active coping (AC)	W	10.90	2.80	-0.42	0.674	0.06
	M	11.06	2.67			
	Total	10.96	2.74			
Use of instrumental social support (ISS)	W	10.68	2.94	2.20	0.030	0.31
	M	9.65	3.41			
	Total	10.29	3.16			
Suppression of competing activities (SCA)	W	9.53	2.69	-0.42	0.676	0.06
	M	9.69	2.82			
	Total	9.59	2.73			
Focus on and venting of emotions (FVE)	W	9.96	3.22	3.47	0.001	0.48
	M	8.41	2.91			
	Total	9.37	3.19			
Mental disengagement (MD)	W	9.33	2.24	1.83	0.069	0.26
	M	8.73	2.34			
	Total	9.10	2.29			
**Perfectionism^2^**						
Striving for perfection (SP)	W	4.45	1.31	-0.06	0.950	0.09
	M	4.46	1.65			
	Total	4.45	1.25			
Negative reactions to imperfection (NRI)	W	3.58	1.31	2.06	0.040	0.29
	M	3.19	1.31			
	Total	3.43	1.32			
Concerns over mistakes and doubts about actions (CMD)	W	2.46	0.87	0.55	0.582	0.08
	M	2.39	0.89			
	Total	2.43	0.88			
**Sleep quality (PSQI)^2^**	W	5.39	2.81	0.725	0.470	0.10
	M	5.13	2.24			
	Total	5.29	2.60			
**Fatigue^2^**	W	13.36	4.51	0.68	0.496	0.09
	M	12.95	3.69			
	Total	13.20	4.21			


*W, women; M, men. ^*1*^*N* = 483, Women *n* = 286, Men *n* = 197, *df* = 482. ^*2*^*N* = 205, Women *n* = 127, Men *n* = 78, *df* = 204. Different values for degrees of freedom (*df*) reflect completion of individual scales.*

### Wellbeing

**FIGURE 2:**
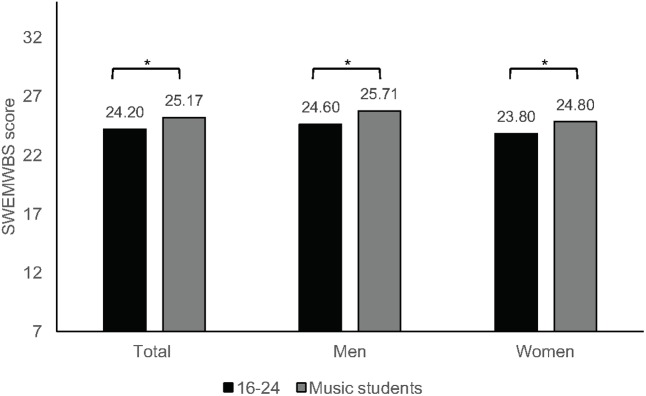
Mean scores for wellbeing (SWEMWBS) for music students and from population data (^∗^*p* ≤ 0.001).

The mental wellbeing of music students was compared against mean values of the SWEMWBS raw score published for the United Kingdom population (Office for National Statistics, 2016) using one sample *t*-tests. Music students scored significantly higher than then general population overall (*t*_482_ = 3.435, *p* = 0.001, *d* = 0.31); women (24.8 ± 3.6) did not differ from women’s scores in the population study (*M* = 24.47, *t*_285_ = 1.577, *p* = 0.116, *d* = 0.19), but men (25.7 ± 3.5) scored significantly higher compared with all men in the population study (*M* = 24.75, *t*_196_ = 3.774, *p* < 0.001, *d* = 0.54). Music students showed higher mental wellbeing when compared with people aged 16–24 and 25–34 (*t*_482_ = 5.941, *p* < 0.001, *d* = 0.54) (**Figure 2**). Data for different age groups by sex is available only for young people aged 16- to 24-years-old, and both women (*M*_16-24_ = 23.8, *t*_285_ = 4.740, *p* < 0.001, *d* = 0.56) and men (*M*_16-24_ = 24.6, *t*_196_ = 4.367, *p* < 0.001, *d* = 0.62) showed significantly higher scores than their peers.

For the following within-sample analysis, we used the metric score as the outcome variable, as recommended by Stewart-Brown et al. (2009), as it allows appropriate distribution of the raw scores. After entering each background variable (sex, level of study, and instrument group) on a hierarchical multiple linear regression procedure, wellbeing was best explained by sex only (*F*_1,481_ = 7.822, *p* = 0.005), with *R*^2^ = 0.016 and adjusted *R*^2^ = 0.012. These results show that women tend to have lower wellbeing scores than men (*B* = -0.773, β = -0.126, *t* = -2.797, *p* = 0.005, CI [-1.32, -0.23]), which has been observed in previous studies (Booker and Sacker, 2011; Davoren et al., 2013; Office for National Statistics, 2016).

Overall, music students have high levels of wellbeing compared with normative data, with expected differences between women and men. Studies with university level students (Davoren et al., 2013) have shown similar patterns of results in terms of sex differences as observed in population studies. However, The Office for National Statistics does not provide comparable data for students in higher education, only for approximately similar age groups, and therefore, comparisons should be interpreted with caution. Nevertheless, these findings suggest that music students—and particularly male students—perceive themselves as psychologically well and fully functioning. Wellbeing is crucial for maintaining motivation to learn, positive social relationships and commitment (Ryan and Deci, 2000); thus, it is essential for music students to find environments and opportunities that foster personal growth and psychosocial wellbeing.

### General Health

In the present study, the perceived general health mean score was 61.83 ± 17.85 on a scale of 0–100 where higher values indicate better self-rated health. When compared with published norms in the United Kingdom for tertiary education students using one sample *t*-tests with mean values (Brazier et al., 1992; Jenkinson et al., 1999), musicians’ perceptions of health were significantly lower, both for women and men with high effect sizes ranging from 1.06 to 1.59 (see Supplementary Table 2). Comparisons with more recent studies with university students in the United Kingdom (Roberts et al., 2000; Stewart-Brown et al., 2000) revealed significantly poorer results for music students than have been reported previously (Steinmetz et al., 2012) as shown in Supplementary Table 2.

Previous research reveals differences between women and men on self-rated health (McDowell, 2006). However, hierarchical regression models in our study showed that sex, level of study and instrument group did not contribute to a working predictive model of self-rated health of music students.

While university level students tend to report their health as poor, it is concerning that music students report even worse health than their peers. These results indicate that music students have low expectations and evaluations regarding their general health which, based on previous literature, may reflect poor health status and influence their quality of life. Jylhä (2009) proposes that perceptions and self-assessment of health depend on individuals’ knowledge of health information and interactions with the environment through previous experiences and peer comparisons. As reported by Perkins et al. (2017), music students acknowledge the importance of good health but also comment on the low priority given to health matters in the conservatoire environment. Therefore, it is relevant to explore how educators and conservatoires can contribute more effectively to more positive perceptions of health.

### Health-Promoting Behaviors

When compared with normative data using the original scale (Walker et al., 1988), differences were observed between the overall score, HR, IR, SG, and SM, with significantly lower scores in our sample of music students (Supplementary Table 3). Additional comparisons were made with the findings of previous studies of musicians and non-musicians of similar ages using one-sample *t*-tests and published mean values (Divin, 2009; Kreutz et al., 2009; Wei et al., 2012; Panebianco-Warrens et al., 2015). Significant differences were observed for most subscales, although with inconsistent patterns. High effect sizes (Cohen’s *d* between 0.59 and 1.92) were observed for SM scores, generally with lower scores observed in our sample. However, no significant differences were found in the overall score when comparing our sample with music students and other university students of similar ages, which indicate a similar, and irregular, pattern of health awareness and behaviors across students in tertiary education that seems typical of this age group (Laidlaw et al., 2016).

**FIGURE 3:**
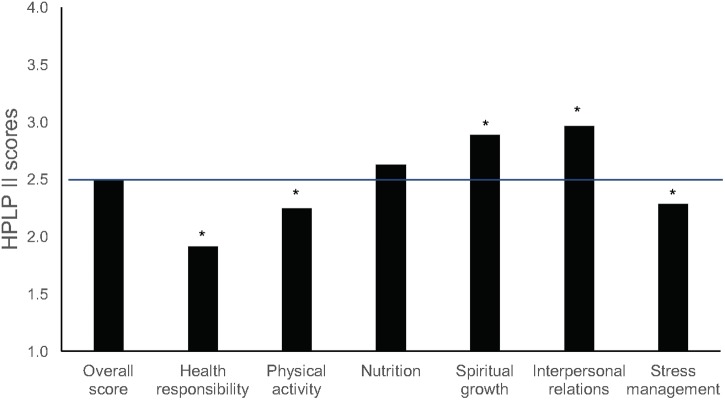
Mean scores for health-promoting behaviors (HPLP II) for music students, including the HPLP II overall score and individual subscale scores. The horizontal line indicates the scale mid-point of 2.5 (^∗^*p* ≤ 0.001).

In our study, the mean score overall for health-promoting behaviors for music students was near the mid-point of the scale (2.5 ± 0.34), suggesting that overall they engage *sometimes* or *often* in health-promoting behaviors. An analysis of the mean values across subscales (**Table 2**) using one-sample *t*-tests suggests that most scores are within the category *sometimes*, which indicates sufficient levels of engagement. Scores were significantly higher than the mid-point of 2.5 (*p* < 0.001) for IR (2.97 ± 0.48) and SG (2.89 ± 0.49), dimensions that involve a sense of connectedness and belonging, as well as NU (2.63 ± 0.52), showing that music students engage in healthy eating with some regularity (**Figure 3**). The lowest scores, where means were significantly less than 2.5 (*p* < 0.001), were observed for HR (1.92 ± 0.5), SM (2.29 ± 0.42), and PA (2.25 ± 0.58). These results indicate low levels of proactive engagement in behaviors related to seeking professional help and looking after their health (HR), mobilizing physical and psychological resources to control stress (SM), and engaging regularly in physical activity (PA).

Hierarchical multiple regression analysis (**Table 3**) showed that sex, level of study and instrument group did not predict overall engagement of music students in health-promoting behaviors. However, significant effects for level of study (postgraduate) were found on HR, showing that studying at postgraduate level is associated with higher levels of health responsibility. Results also showed that women have higher engagement in healthy eating and social interactions than men, as found in previous studies (Stock et al., 2001; Von Bothmer and Fridlund, 2005; Divin, 2009; Wei et al., 2012; Panebianco-Warrens et al., 2015).

**Table 3:** Hierarchical multiple regression analysis of independent baseline predictors of health-promoting behaviors (HPLP II).

	HPLP II	HR	PA	NU	IR	SG	SM
	β	β	β	β	β	β	β
**Model 1**							
Sex	0.057	0.049	-0.051	0.163^‡^	0.174^‡^	-0.049	-0.079
*R*^2^, Adjusted *R*^2^	0.003, 0.001	0.002, 0.000	0.003, 0.000	0.027, 0.025^‡^	0.030, 0.028^‡^	0.002, 0.000	0.006, 0.004
**Model 2**							
Sex	0.049	0.029	-0.045	0.170^‡^	0.166^‡^	-0.061	-0.084
Level of Study	-0.055	-0.143^‡^	0.042	0.048	-0.052	-0.088	-0.039
*R*^2^, Adjusted *R*^2^	0.006, 0.002	0.023, 0.018^†^	0.004, 0.000	0.029, 0.025^‡^	0.033, 0.028^‡^	0.010, 0.006	0.008, 0.004
**Model 3**							
Sex	0.029	0.011	-0.067	0.165^‡^	0.158^‡^	-0.077	-0.098*
Level of study	-0.037	-0.119*	0.068	0.041	-0.040	-0.082	-0.026
Keyboard	-0.083	-0.011	-0.076	-0.120	-0.064	-0.046	-0.004
Woodwind	-0.010	-0.002	0.035	-0.031	0.000	-0.060	0.024
Brass	-0.062	-0.023	0.016	-0.078	-0.045	-0.104*	-0.010
Voice	0.070	0.114*	0.136^‡^	-0.069*	0.045	0.017	0.046
Percussion	-0.033	-0.027	-0.021	-0.043	0.016	-0.017	-0.047
Other	0.015	-0.010	-0.014	0.047	0.043	0.008	-0.014
*R*^2^, Adjusted *R*^2^	0.023, 0.007	0.037, 0.021^∗^	0.033, 0.016^∗^	0.050, 0.034^†^	0.044, 0.028^‡^	0.023, 0.007	0.013, -0.004


*Sex = Female, Level of Study = Undergraduate. *N* = 483, ^∗^*p* < 0.05, ^†^*p* < 0.01, ^‡^*p* ≤ 0.001.*

Our results confirm previous findings that show poor engagement in health-promoting behaviors among music students (Kreutz et al., 2008, 2009; Panebianco-Warrens et al., 2015). The comparative results for each dimension of HPLP II (as reported in Supplementary Table 3) also suggest that, among tertiary students of comparable age, there is wide variation across domains of study. As to subscales of HPLP II, higher scores in IR and SG compared with other subscales have been reported in previous studies with musicians and non-musicians (Kreutz et al., 2008, 2009; Divin, 2009; Peker et al., 2010; Wei et al., 2012; Panebianco-Warrens et al., 2015). This may be explained by the fact that, during their time at college or university, students develop in their daily routines and in interaction with peers a sense of belonging, connectedness, and direction toward the future (Laidlaw et al., 2016). In line with the discussion above of wellbeing, music students seem to find opportunities to build an effective and supportive social network, as well as a sense of growth and purpose in life.

However, considering the demands on musicians in both educational and professional settings and the evidence on prevalence of mental and physical health problems, the levels of engagement in PA, SM, and HR among music students are disconcertingly low. The effects of poor lifestyle habits on learning and performance can be considerable and appear to be underestimated generally by musicians, as suggested by our results and those of previous studies over the past 10 years (Kreutz et al., 2008, 2009; Panebianco-Warrens et al., 2015). Action is needed to understand better why these behaviors are particularly lacking among musicians and the sources of impediment to them. Research suggests that engagement in healthy behaviors typically results from interactions between an individual and his or her environment (Pender, 2011); as such, those who train and employ musicians play a crucial role in developing healthy settings and fostering positive attitudes to health.

### Coping

Compared with the validation study of the COPE scale (Carver et al., 1989), where 117 undergraduate students completed a situational version of the inventory, one-sample *t*-tests using published mean values showed significant differences (*p* ≤ 0.001) with medium to large effect sizes (*d* = 0.49–0.81) on P, AC, use of ISS, and FVE with lower scores observed overall in our study (Carver et al., 1989) (Supplementary Table 4). As mentioned previously, the MD scale was not used due to poor internal validity.

The most frequently used coping strategies, based on mean values, PRG (12.1 ± 2.35), P (11.09 ± 2.91), AC (11.06 ± 2.67), and use of ISS (10.29 ± 3.16). PRG involves reframing the stressor in positive ways, P involves generating a plan of action to deal with a stressor, and AC involves taking action to remove or deal with a stressor. The less used coping strategies were FVE (9.37 ± 3.19) and SCA (9.59 ± 2.73).

Our finding that PRG, P, AC, and ISS are used most by music students corresponds with results of previous studies with different samples (Grove et al., 1997; Kallasmaa and Pulver, 2000; Litman and Lunsford, 2009). For example, Litman and Lunsford (2009) grouped all the COPE scales into three dimensions: *self-sufficient approach-oriented* coping (including PRG, AC, P, SCA), *socially supported approach-oriented* coping (including ISS, FVE), and *avoidant-oriented* coping approach (including MD). In their study with 450 university students they found that self-sufficient approach-oriented coping strategies were the most frequently used. Our findings indicate a similar pattern of usage of coping strategies, yet their overall use was poor.

Hierarchical multiple regression analysis (**Table 4**) showed that sex, level of study, and instrument group best predicted ISS (6%) and FVE (7%) when fitted together, as well as SCA (4%) but with a marginal significance. Use of ISS and FVE seems to be consistently predicted by sex (being a woman) and level of study (postgraduate), suggesting that women studying at postgraduate level tend to use more coping strategies related to ISS and venting of emotions.

**Table 4:** Hierarchical multiple regression analysis of independent baseline predictors of coping (COPE Inventory).

	COPE PRG	COPE P	COPE AC	COPE ISS	COPE SCA	COPE FVE
	β	β	β	β	β	β
**Model 1**						
Sex	-0.036	0.082	-0.030	0.158*	-0.029	0.237^‡^
*R*^2^, Adjusted *R*^2^	0.001, -0.004	0.007, 0.002	0.001, -0.004	0.005, 0.020^∗^	0.001, -0.004	0.056, 0.051^‡^
**Model 2**						
Sex	-0.039	0.080	-0.034	0.149*	-0.031	0.229^‡^
Level of Study	-0.067	-0.036	-0.083	-0.174*	-0.035	-0.149*
*R*^2^, Adjusted *R*^2^	0.006, -0.004	0.008, -0.002	0.008, -0.002	0.055, 0.046^†^	0.002, -0.008	0.078, 0.069^‡^
**Model 3**						
Sex	-0.077	0.076	-0.034	0.120	-0.083	0.189^‡^
Level of study	-0.055	-0.025	-0.078	-0.164*	-0.050	-0.125
Keyboard	0.044	0.098	0.157*	0.173*	0.105	-0.006
Woodwind	-0.008	-0.072	0.008	0.123	0.020	0.081
Brass	-0.066	-0.025	0.064	0.070*	-0.105	0.024
Voice	0.074	0.037	0.064	0.153	-0.071	0.151
Percussion	-0.066	-0.113	-0.104	-0.025	-0.190*	-0.044
Other	-0.074	0.051	0.053	-0.050	-0.126	-0.038
*R*^2^, Adjusted *R*^2^	0.028, -0.012	0.044, 0.005	0.047, 0.008	0.098, 0.061^†^	0.081, 0.043^∗^	0.105, 0.069^†^


*Sex = Female, Level of Study = Undergraduate. *N* = 205, ^∗^*p* < 0.05, ^†^*p* < 0.01, ^‡^*p* ≤ 0.001.*

Kallasmaa and Pulver (2000) reported a similar pattern of results in those scales (ISS and FVE) in a study with undergraduate students, although they found significant sex differences across most subscales. The evidence for sex differences in coping is inconsistent, and although some studies suggest that women tend to use more emotional coping and men use more problem-focused coping, that was not observed in our findings (Matud, 2004; Wilson et al., 2005; Kelly et al., 2008; Helgeson, 2011; Doron et al., 2014). Coping strategies are central to dealing effectively with stressful events, and it has been reported that flexibility in coping is a key characteristic of world-class musicians (MacNamara et al., 2008). It is, therefore, concerning that music students’ use of coping strategies is limited and that so little attention has been given to this in both research and music training.

### Perfectionism

When compared with previous studies using one sample *t*-tests with published mean values (Supplementary Table 5), in particular a study with younger musicians (Stoeber and Eismann, 2007), our sample scored significantly higher in SP (*t*_204_ = 5.40, *p* < 0.001, *d* = 0.76). When compared with a sample of athletes of similar age (Stoeber et al., 2007), no significant differences were found. This suggests that perfectionistic tendencies develop along with increasing levels of expertise and develop as a characteristic of elite performers. Following the recommendations by Stoeber and Eismann (2007), additional measures addressing concerns over mistakes and doubts about actions were used. Music students showed average levels of CMD (2.43 ± 0.88 in a range of 1–5), and no significant differences were found when compared with Stoeber’s study of students of similar age using a one sample *t*-test (Stoeber, 1998).

Music students’ mean scores on SP were 4.45 (±1.25) in a scale ranging from 1 (never) to 6 (always), showing a tendency to frequent feelings of perfectionistic strivings. They showed less frequent feelings of negative reactions to imperfection (NRI = 3.43 ± 1.32). As shown in **Table 5**, hierarchical multiple regression analysis revealed that perfectionistic tendencies were best predicted by model 2, where level of study (undergraduate) was a major predictor of all dimensions of perfectionism. Sex was only predictive of NRI, suggesting that women are more disposed to react negatively to mistakes.

**Table 5:** Hierarchical multiple regression analysis of independent baseline predictors of perfectionism.

	SP	NRI	CMD
	β	β	β
**Model 1**			
Sex	-0.004	0.143*	0.039
*R*^2^, Adjusted *R*^2^	0.000, -0.005	0.021, 0.016^∗^	0.001, -0.003
**Model 2**			
Sex	0.007	0.153*	0.051
Level of study	0.234^‡^	0.197^‡^	0.242^‡^
*R*^2^, Adjusted *R*^2^	0.054, 0.045^†^	0.059, 0.050^†^	0.060, 0.051^†^
**Model 3**			
Sex	0.016	0.200^‡^	0.085
Level of study	0.259^‡^	0.193^‡^	0.252^‡^
Keyboard	-0.115	-0.007	-0.037
Woodwind	-0.036	-0.097	-0.089
Brass	0.034	0.087	0.020
Voice	0.073	0.012	0.023
Percussion	-0.006	0.076	0.069
Other	0.100	0.066	0.088
*R*^2^, Adjusted *R*^2^	0.089, 0.052^∗^	0.088, 0.051^∗^	0.084, 0.046^∗^


*Sex = Female, Level of study = Undergraduate. *N* = 205, ^∗^*p* < 0.05, ^†^*p* < 0.01, ^‡^*p* ≤ 0.001.*

Previous research has found limited and mixed evidence for sex differences in perfectionism, and only in relation to academic performance (Kawamura et al., 2002; Blankstein and Winkworth, 2004). As stated by Stoeber (2012), most studies do not report sex differences, and little is known about such differences in perfectionism. The perfectionistic tendencies observed here indicate that music students are highly driven to succeed, especially during their undergraduate studies. It may be that perfectionistic tendencies develop alongside musical training as a characteristic of elite musicians. However, in such a competitive setting, these students need to develop mechanisms to moderate the high expectations they face (self-directed and from others) at early stages of their careers, which can lead to increased levels of stress, disappointment, and frustration, before these develop into maladaptive forms of perfectionism.

### Sleep Quality

The mean score of the PSQI was 5.29 (±2.60), and a one sample *t*-test showed no significant differences when compared with the recommended cut-off point of 5 for risk of sleep disturbances (*t*_204_ = 1.58, *p* = 0.115, *d* = 0.22) (Buysse et al., 1989). When compared with findings from the validation study (Supplementary Table 6) (Buysse et al., 1989), music students reported significantly poorer sleep (*t*_204_ = 14.75, *p* < 0.001, *d* = 2.1). Buysse et al. (1989) found no correlation of the PSQI score with age but the mean age in the validation study was 59.9-years-old. Therefore, comparisons should be interpreted cautiously.

Comparisons with other population studies using one sample *t*-tests separately by sex with available mean scores (Supplementary Table 6) showed that music students reported better sleep quality overall, both for women and men, than similar age groups (Lund et al., 2010; Orzech et al., 2011; Chang et al., 2016).

Hierarchical multiple regression analysis showed that sex, level of study and instrument group did not contribute to a working model to predict sleep quality. While these findings suggest that music students have better sleep than their peers in other areas of study, the results still show borderline and worse scores than the general population. The benefits of good sleep habits for psychological health, learning, and performance are well documented but currently do not feature as part of musicians’ training. In addition, a good night’s sleep may be difficult to achieve for many, due to musicians’ busy schedules, late working hours, and constant pressure to excel.

### Fatigue

Fatigue levels of music students in this sample were low overall (13.2 ± 4.21 of a maximum possible score of 33, where higher scores indicate high levels of fatigue). A one sample *t-test* using mean scores of a United Kingdom community sample (*N* = 1615, age *M* = 34 ± 7.6, fatigue score *M* = 14.2) showed significant differences and medium effect sizes (*t*_204_ = 3.382, *p* = 0.001, *d* = 0.47), with music students reporting lower levels of fatigue (Cella and Chalder, 2010). Data for women and men were not available for comparisons. These findings seem to suggest that, despite the high intensity and competitiveness of their activities, music students still feel energetic, concentrated, and cognitively active. However, Cella and Chalder’s (2010) sample average age was 34-years-old (*SD* = 7.6); therefore, comparisons should be interpreted cautiously.

Similar to that observed for sleep quality, baseline independent descriptors (sex, level of study and instrument group) did not contribute to a working regression model, and thus, these variables seem to have no predictive association with fatigue.

## Conclusion

Our study investigated music students’ health and wellbeing and extends previous literature by providing a comprehensive picture of their health-related perceptions, attitudes, and behaviors in comparison with similar samples. In some respects, the health profile of music students presented here follows typical patterns seen among other tertiary students, but these similarities are not necessarily a positive sign of good health, especially for a group of specialist students who are distinctive from others in higher education in terms of the acute physical and mental stress they face during training and the uncertain and highly competitive professional landscape they are preparing to enter.

Some positive results emerged with our findings, showing that music students on average engage at adequate levels in health-promoting behaviors related to social and spiritual dimensions, they score high on wellbeing, and they display low levels of fatigue, which may contribute to optimal psychological health and functioning. It is particularly intriguing that musicians have high levels of wellbeing despite the high prevalence of pain, injury, and anxiety often reported in the literature (Ascenso et al., 2017; Cruder et al., 2017), and it would be relevant to investigate further how wellbeing in musicians changes over time and in relation to particular challenges and obstacles faced at different career stages. Yet, their other perceptions, attitudes, and behaviors toward health are less than optimal.

Limited engagement in regular physical activity and low self-rated health suggest that music students’ overall health status is poor. This raises some concerns, in particular with regards to how music students’ lifestyle and perceptions of health impact on the way they engage with music learning and performance. Although sleep quality of music students was not at the level of clinical disorder, the overall score was poor when compared with the general population. Sleep has an important restorative function with impact on memory and learning, and thus it is relevant to investigate the sleep practices and sleep quality of musicians, as well as its the specific impact on music learning and performance; a good night’s sleep may be difficult to achieve for many, due to their busy schedules, late working hours, and constant pressure to excel. The potential impact of performance training on music students’ sleep quality, fatigue, and physical health, and vice-versa, remain yet to be fully investigated; this is an important consideration as students in educational settings may seek (or be offered) 24-h availability of practice rooms. Additionally, the benefits of regular physical activity to prevent physical ill-health and promote psychological health are well known and would seem particularly important for musicians considering the physical and psychological demands they face. However, music students’ engagement in regular physical activity as a health-promoting behavior is low. Research on musicians’ fitness is limited, and therefore, it would be instructive to explore the physical readiness of music students and to monitor their levels of engagement in regular physical activity in order to understand better the specific impact of lifestyle behaviors related to regular physical activity on their health and wellbeing and on their performance.

Similarly, music students’ engagement in health responsibility and stress management behaviors is low which, along with limited use of coping strategies and high perfectionistic strivings, generates an alarming mental health forecast. This is concerning in a field that is characterized by constant high pressure and competitiveness. Therefore, the need for psychological health education and intervention—driven by both individuals and educational institutions—from early career stages is urgent and should be considered proactively, before health problems arise. Instead, most interventions and initiatives for physical and psychological health in conservatoire settings still develop as a result of identified problems that need fixing (e.g., MPA, musculoskeletal problems, and pain) rather than focusing on equipping students with the skills necessary to prevent, understand, and deal with the challenges of music making. It is imperative that those in specialist music education communities where these students develop (including parents, teachers, administrators, managers, and other support staff) commit collectively to the development of mechanisms that support students to build psychological resilience to achieve optimal health and wellbeing and optimize their practice and performance. As an example, in 2015, the Healthy Conservatoires Network^2^ was established using whole-system and setting-based approaches in order to address some of the issues related to performers’ health and wellbeing by encouraging different players in the conservatoire setting to discuss, and engage with, young performers’ health and wellbeing. Embedding and supporting health awareness as part of the curriculum, offering professional development activities on health education to instrumental teachers, and make health screening initiatives available are some examples of how specialist music education institutions can contribute to the development of healthier musicians from early ages.

Our results should be interpreted in the light of some limitations, which also give rise to several avenues for further investigation. First, the sample consisted of music students from several conservatoires in the United Kingdom and Switzerland with assorted representation by institution, geographical area, and country of origin. Cultural, local, and institutional experiences undoubtedly influence the way people think about and evaluate their own health and wellbeing (Steptoe and Wardle, 2001; Wardle et al., 2004; Jylhä, 2009). While it remains relevant to investigate the uniqueness of institutions in promoting individuals’ health and wellbeing, it is also important to explore patterns of perceptions, attitudes, and behaviors toward health at an international level with cross-cultural representation. Second, participants self-selected to take part in this study, and so, our results reflect the health profiles of a particular sample. It remains to be seen whether music students who did not (or were not willing to) take part present a similar profile. Given the somewhat mixed picture of health seen in this sample—all of whom were aware of the aims of the study and showed enough interest in their health to take part—a fully comprehensive picture may reveal a somewhat bleaker picture toward health among the wider population of music students. Third, our results are based on self-report measures that were part of a long screening protocol, which may have resulted in answers flavored by social desirability or fatigue. Well-established, standardized measures were used, and the approach is not dissimilar to a multitude of published health promotion studies. Nonetheless, comparing these results with other objective health data could provide valuable information for elucidating links between health perceptions, experiences, and performance. Finally, as a cross-sectional study, our results are confined to students’ experiences and feelings at a specific moment in time and with specific challenges faced at that time. In collecting data, we aimed to avoid particularly busy performance or examination times, and students were asked to recall, in any case, the most recent typical working periods when completing the questionnaires. A longitudinal approach would allow for a clearer picture of the ebb and flow of perceptions, attitudes, and behaviors toward health.

In understanding the potential impact and practical implications of our findings, it seems relevant to address the concept of health literacy (Nutbeam, 2000, 2008). Health literacy is defined as the capacity of individuals to have access to, understand, and use health information to make informed choices about health. In developed societies, this concept involves more than access to information and includes increasing proactivity in handling health-related information and provision. Indeed, access to information is available to these students, but they still need to develop the skills, motivation and confidence to engage critically with and tailor the available information toward their personal needs and benefits. According to Nutbeam (2000, 2008), this progress from a functional level of knowledge to an interactive and critical level of literacy allows for greater autonomy, personal empowerment, and optimal health changes. However, this cannot be the pursuit of individuals alone and requires an active role of communities and institutions. Therefore, conservatoires and music schools have a central role in increasing the levels of individual and institutional health literacy. This can be achieved by developing understanding of health literacy levels of students, by promoting the necessary opportunities for individual and organizational change, and by sustaining a culture that promotes self-agency and behavioral engagement in health matters.

## Author Contributions

All authors contributed extensively to the work presented in this paper.

## Conflict of Interest Statement

The authors declare that the research was conducted in the absence of any commercial or financial relationships that could be construed as a potential conflict of interest.

## Footnotes

1 http://www.healthyuniversities.ac.uk

2 http://www.healthyconservatoires.org
