# Supplementary material for: Fit to Perform: An Investigation of Higher Education Music Students’ Perceptions, Attitudes, and Behaviors toward Health
Source: Front Psychol. 2017 Oct 10;8:1558. doi: 10.3389/fpsyg.2017.01558 (PMC5641399; doi:10.3389/fpsyg.2017.01558)
Supplement: Supplementary file 1 [file Table_1.pdf]

Araújo LS, Wasley D, Perkins R, Atkins L, Redding E, Ginsborg J and Williamon A (2017), Fit to Perform: An Investigation of Higher Education Music Students' Perceptions, Attitudes, and Behaviors toward Health, *Front. Psychol.* 8:1558. doi: 10.3389/fpsyg.2017.01558

SUPPLEMENTARY TABLE 1 | The Fit to Perform protocol. Results reported in this article are drawn from Stage 2, a survey of the perceptions, attitudes, and behaviors of music students toward health and wellbeing.

Stage 1: Briefing, initial screening, and informed consent (5 min)

- Consent form
- Physical Activity Readiness – Questionnaire (PAR-Q)

Stage 2: Survey of perceptions, attitudes, and behaviors toward health (30-40 min)

- Background: Age, sex, current educational/professional status, instrument, genre, and practice/performance behaviors
- Lifestyle behaviors: Smoking, alcohol, and caffeine consumption
- Sleep quality: Pittsburgh Sleep Quality Questionnaire (PSQI; Buysse et al., 1989)
- Wellbeing: Short Warwick Edinburgh Mental Well-being Scale (SWEMWBS; Stewart-Brown et al., 2009)
- Physical activity: International Physical Activity Questionnaire Short-Form (IPAQ-SF; https://sites.google.com/site/theipaq)
- Fatigue: Chalder Fatigue Questionnaire (CFQ; Chalder et al., 1993; Cella & Chalder, 2010)
- Health promoting behaviors: Health Promoting Lifestyle Profile II (HPLP II; Walker & Hill-Polerecky, 1996)
- Coping: COPE Inventory (Carver et al., 1989) subscales 'Positive reinterpretation and growth' (PRG), 'Planning' (P), 'Active coping' (AC), 'Use of instrumental social support' (ISS), 'Suppression of competing activities' (SCA), 'Focus on and venting of emotions' (FVE), and 'Mental disengagement' (MD)
- Perfectionism: Multidimensional Inventory of Perfectionism in Sports (MIPS; Stoeber & Eismann, 2007); Frost Multidimensional Perfectionism Scale (FMPS; Frost et al. 1990) subscales 'Concern over mistakes' and 'Doubts about actions'
- General health: General Health Scale from RAND Short Form 36 Health Survey (SF-36; Ware et al., 1993)
- Performance-related musculoskeletal pain: Digital Pain Drawing (Barbero et al., 2015; Cruder et al., 2017)

Stage 3: Health-related fitness assessment (30-35 min)

- Hand and finger span
- Blood pressure
- Height
- Weight
- Lung capacity
- Hypermobility (Beighton test)
- External and internal rotation of shoulders (Apley test)
- Flexibility: Shoulder reach test
- Posture
- Grip strength test
- Sit and reach test
- Plank test
- Press up test
- Step test
- Performance-related musculoskeletal problems: Nordic Musculoskeletal Questionnaire–Extended (Dawson et al., 2009)
- Musculoskeletal disorders of the upper limbs: Quick Dash, including Performing Arts Module (Institute for Work and Health, 2006)

Stage 4: Feedback (5-10 min)

- Individualized feedback report on Stage 3 fitness results, as well as relevant health-promotion leaflets and health education information
