# Supplementary material for: Fit to Perform: An Investigation of Higher Education Music Students’ Perceptions, Attitudes, and Behaviors toward Health
Source: Front Psychol. 2017 Oct 10;8:1558. doi: 10.3389/fpsyg.2017.01558 (PMC5641399; doi:10.3389/fpsyg.2017.01558)
Supplement: Supplementary file 2 [file Table_2.pdf]

Araújo LS, Wasley D, Perkins R, Atkins L, Redding E, Ginsborg J and Williamon A (2017), Fit to Perform: An Investigation of Higher Education Music Students' Perceptions, Attitudes, and Behaviors toward Health, *Front. Psychol.* 8:1558. doi: 10.3389/fpsyg.2017.01558

SUPPLEMENTARY TABLE 2 | Means (standard deviations) for self-rated general health (RAND SF-36) for the current study and previous research with population and students samples.

Domain	N=205	Brazier et al. (1992) N=1,582		Stewart et al. (2000) N=1,208		Roberts et al. (2000) N=482	
	Music	Diverse		Diverse		Diverse	
	M (SD)	Range	$t_{[1]\ [2]\ [3]}$, d	M	t_{204}, d	M	t_{204}, d
Age	21.30 (3.64)	16-24		23 (21)		23.4 (4.8)	
General Health							
Women	60.98 (18.57)	71[4]	-6.08, 1.08‡	-	-	-	-
Men	63.20 (16.63)	72[5]	-4.67, 1.06‡	-	-	-	-
Total	61.83 (17.85)	76[6]	-11.37, 1.59‡	66.01[7]	-3.35, 0.47‡	66.33 (20.89)	-3.61, 0.51‡

Note. [1] *df* = 126, [2] *df* = 77, [3] *df* = 204, [4] n = 829 and refers to the total number of women of all ages in the entire sample (N = 1,582) who completed the scale, [5] n = 675 and refers to the total number of men of all ages in the entire sample (N = 1,582) who completed the scale, [6] n = 240 and refers to total number of participants aged 16-24 (similar to the current study) in the entire sample (N = 1,582) who completed the scale, [7] median score. M(*SD*)= Mean (standard deviation), *d*= Cohen's d, RAND SF-36 = RAND Short Form 36 Health Survey. Significant differences between previous studies and the current study indicated by ‡$p \leq 0.001$.
