# Supplementary material for: Fit to Perform: An Investigation of Higher Education Music Students’ Perceptions, Attitudes, and Behaviors toward Health
Source: Front Psychol. 2017 Oct 10;8:1558. doi: 10.3389/fpsyg.2017.01558 (PMC5641399; doi:10.3389/fpsyg.2017.01558)
Supplement: Supplementary file 3 [file Table_3.pdf]

Araújo LS, Wasley D, Perkins R, Atkins L, Redding E, Ginsborg J and Williamon A (2017), Fit to Perform: An Investigation of Higher Education Music Students' Perceptions, Attitudes, and Behaviors toward Health, *Front. Psychol.* 8:1558. doi: 10.3389/fpsyg.2017.01558

SUPPLEMENTARY TABLE 3 | Means (standard deviations) for health promoting behaviors (HPLP II) for the current study and previous research with music and non-music students.

	**N=483**	**Walker et al. (1988) N=167[1]**		**Kreutz et al. (2009) N=269**		**Panebianco et al. (2014) N=146**		**Divin (2008) N=98**		**Wei et al. (2012) N=336**		**Peker & Bermek (2010) N=165**	
**Domain**	Music	Diverse		Music		Music		Sports		Diverse		Dentistry	
	M (SD)	M (SD)	t_{482}, d	M (SD)	t_{482}, d	M (SD)	t_{482}, d	M (SD)	t_{482}, d	M (SD)	t_{482}, d	M (SD)	t_{482}, d
Age	21.3 (3.60)	Range: 18-34		21.9 (3.20)		Range: 18-37		19.7 (1.66)		20.1 (1.5)		19.4 (0.89)	
HPLP II Overall	2.50 (0.34)	2.63 (0.40)	-8.54, 0.78‡	2.47 (0.40)	1.79, 0.16	2.59 (0.36)	-5.96, 0.54‡	2.41 (0.46)	5.67, 0.52‡	2.50 (0.29)	-0.15, 0.01	2.49 (0.32)	0.50, 0.04
HR	1.92 (0.50)	2.00 (0.52)	-3.61, 0.33‡	1.81 (0.52)	4.74, 0.43‡	2.01 (0.52)	-4.05, 0.37‡	1.89 (0.48)	1.22, 0.11	2.01 (0.53)	-4.05, 0.37‡	2.12 (0.48)	-8.88, 0.81‡
PA	2.25 (0.58)	2.22 (0.78)	1.07, 0.10	2.22 (0.63)	1.07, 0.10	2.39 (0.62)	-5.32, 0.48‡	2.71 (0.62)	-17.36, 1.58‡	2.23 (0.59)	0.69, 0.06	2.16 (0.59)	3.33, 0.30‡
NU	2.62 (0.52)	2.59 (0.67)	1.50, 0.14	2.71 (0.60)	-3.57, 0.25‡	2.57 (0.54)	2.34, 0.21*	2.31 (0.58)	13.31, 1.21‡	2.42 (0.49)	8.67, 0.79‡	2.14 (0.42)	20.49, 1.87‡
IR	2.97 (0.48)	3.16 (0.51)	-8.87, 0.81‡	2.95 (0.54)	0.74, 0.07	3.11 (0.50)	-6.52, 0.59‡	2.60 (0.65)	16.76, 1.53‡	3.05 (0.44)	-3.83, 0.35‡	2.91 (0.46)	2.57, 0.23†
SG	2.88 (0.49)	3.10 (0.50)	-9.65, 0.88‡	2.88 (0.56)	0.25, 0.02	3.15 (0.50)	-11.89, 0.08‡	2.76 (0.65)	5.64, 0.51‡	2.61 (0.48)	12.38, 1.13‡	3.03 (0.41)	-6.50, 0.59‡
SM	2.29 (0.42)	2.47 (0.48)	-9.42, 0.86‡	2.26 (0.49)	1.69, 0.15	2.47 (0.49)	-9.42, 0.86‡	2.17 (0.49)	6.45, 0.59‡	2.69 (0.44)	-21.05, 1.92‡	2.52 (0.38)	-11.52, 1.05‡

Note. [1] Data collected with the first version of HPLP. M (SD) = Mean (standard deviation), d = Cohen's d, HPLP II = Health Promoting Lifestyle Profile II, HR = Health responsibility, PA = Physical activity, NU = Nutrition, IR = Interpersonal relations, SG = Spiritual growth, SM = Stress management. Significant differences between previous studies and the current study indicated by * $p<0.05$, † $p<0.01$, ‡ $p\leq0.001$.
