# Supplementary material for: Fit to Perform: An Investigation of Higher Education Music Students’ Perceptions, Attitudes, and Behaviors toward Health
Source: Front Psychol. 2017 Oct 10;8:1558. doi: 10.3389/fpsyg.2017.01558 (PMC5641399; doi:10.3389/fpsyg.2017.01558)
Supplement: Supplementary file 4 [file Table_4.pdf]

Araújo LS, Wasley D, Perkins R, Atkins L, Redding E, Ginsborg J and Williamon A (2017), Fit to Perform: An Investigation of Higher Education Music Students' Perceptions, Attitudes, and Behaviors toward Health, *Front. Psychol.* 8:1558. doi: 10.3389/fpsyg.2017.01558

SUPPLEMENTARY TABLE 4 | Means (standard deviations) for coping (COPE Inventory) for the current study and the COPE validation study by Carver et al. (1989).

	**N=205**	**Carver et al. (1989) N=117**		
	M (SD)	M (SD)	t_{204}	Cohen's d
Positive reinterpretation and growth (PRG)	12.10 (2.35)	12.40 (2.42)	-1.84	0.26
Planning (P)	11.40 (2.92)	12.58 (2.66)	-5.81‡	0.81
Active coping (AC)	10.96 (2.74)	11.89 (2.26)	-4.85‡	0.68
Use of instrumental social support (ISS)	10.29 (3.16)	11.50 (2.88)	-5.50‡	0.77
Suppression of competing activities (SCA)	9.59 (2.73)	9.92 (2.42)	-1.73	0.24
Focus on and venting of emotions (FVE)	9.37 (3.19)	10.17 (3.08)	-3.59‡	0.50

Note. M (SD) = Mean (standard deviation). Significant differences between Carver et al. (1989) and the current study indicated by ‡ $p \leq 0.001$.
