# Supplementary material for: Fit to Perform: An Investigation of Higher Education Music Students’ Perceptions, Attitudes, and Behaviors toward Health
Source: Front Psychol. 2017 Oct 10;8:1558. doi: 10.3389/fpsyg.2017.01558 (PMC5641399; doi:10.3389/fpsyg.2017.01558)
Supplement: Supplementary file 5 [file Table_5.pdf]

Araújo LS, Wasley D, Perkins R, Atkins L, Redding E, Ginsborg J and Williamon A (2017), Fit to Perform: An Investigation of Higher Education Music Students' Perceptions, Attitudes, and Behaviors toward Health, *Front. Psychol.* 8:1558. doi: 10.3389/fpsyg.2017.01558

SUPPLEMENTARY TABLE 5 | Means (standard deviations) for perfectionism for the current study and previous research with music and non-music students.

		**Stoeber & Eismann (2007)**		**Stoeber et al. (2009)**		**Stoeber (1998)[1]**	
	**N=205**	**N=146**		**N=147**		**N=243**	
**Domain**	Music	Music		Sports		Diverse	
	M (SD)	M (SD)	t_{204}, d	M (SD)	t_{204}, d	M (SD)	t_{204}, d
Age	21.9 (3.55)	16.2 (1.80)		22.8 (3.00)		26.3 (5.70)	
Striving for perfection (SP)	4.45 (1.25)	3.98 (1.37)	5.40, 0.76‡	4.52 (1.23)	-0.78, 0.10	-	
Negative reactions to imperfections (NRI)	3.43 (1.32)	3.29 (1.20)	1.53, 0.21	3.26 (1.05)	1.85, 0.26	-	
Concerns over mistakes and doubts about actions (CMD)	2.43 (0.88)	-		-		2.35	1.31, 0.18

Note. [1] Stoeber (1998) reports the sum of scores (M = 30.58, SD = 9.31) on CMD, but for comparative analyses, we calculated the mean by dividing the sum by the total of items. M (SD) = Mean (standard deviation), d = Cohen's d. Significant differences between previous studies and the current study indicated by ‡ p≤0.001.
