# Supplementary material for: Fit to Perform: An Investigation of Higher Education Music Students’ Perceptions, Attitudes, and Behaviors toward Health
Source: Front Psychol. 2017 Oct 10;8:1558. doi: 10.3389/fpsyg.2017.01558 (PMC5641399; doi:10.3389/fpsyg.2017.01558)
Supplement: Supplementary file 6 [file Table_6.pdf]

Araújo LS, Wasley D, Perkins R, Atkins L, Redding E, Ginsborg J and Williamon A (2017), Fit to Perform: An Investigation of Higher Education Music Students' Perceptions, Attitudes, and Behaviors toward Health, *Front. Psychol.* 8:1558. doi: 10.3389/fpsyg.2017.01558

SUPPLEMENTARY TABLE 6 | Means (standard deviations) for sleep quality (PSQI) for the current study and previous research with a community sample (Buysse et al., 1989) and university students.

Domain	N=205	Buysse et al. (1989) N=52		Chang et al. (2016) N=1,230		Lund et al. (2010) N=5,401		Orzech et al. (2011) N=1,823	
	Music	Diverse		Diverse		Diverse		Diverse	
	M (SD)	M (SD)	t_{204}, d	M (SD)	$t_{[1]\,[2]}$, d	M (SD)	$t_{[1]\,[2]}$, d	M (SD)	$t_{[1]\,[2]}$, d
Age	21.30 (3.64)	59.9	-	19.2 (1.10)	1.10	17-24	-	18.6	1.02
PSQI	5.29 (2.60)	2.67 (1.60)	14.40, 2.02‡	-	-	-	-	-	-
Women	5.39 (2.81)	-	-	6.54 (2.70)	$_{[1]}$ -4.64,0.83 ‡	7.2 (3.2)	$_{[1]}$ -7.29, 1.30‡	6.69 (2.79)	$_{[1]}$ -5.24, 0.93‡
Men	5.13 (2.24)	-	-	6.01 (2.81)	$_{[2]}$ -3.48, 0.79‡	6.7 (3.1)	$_{[2]}$ -6.19, 1.41‡	6.38 (2.82)	$_{[2]}$ -4.93, 1.12‡

Note. [1] *df* = 126, [2] *df* = 77. M (SD) = Mean (standard deviation), d = Cohen's d, PSQI = Pittsburgh Sleep Quality Index. Significant differences between previous studies and the current study indicated by † $p<0.01$, ‡ $p\leq0.001$.
